# Modern phenomenology of emotional and cognitive disorders in patients with type II diabetes mellitus

**DOI:** 10.1192/j.eurpsy.2025.2174

**Published:** 2025-08-26

**Authors:** H. Kozhyna, A. Kondratenko, K. Zelenska, I. Tieroshyna

**Affiliations:** 1 Department of Psychiatry, Narcology, Medical Psychology and Social Work, Kharkiv National Medical University, Kharkiv, Ukraine

## Abstract

**Introduction:**

Diabetes mellitus is the most common endocrine disease that occurs as a result of an absolute or relative lack of insulin and is accompanied by a number of different somatic, neurological and mental disorders. The aim of the study is to investigate the clinical, psychopathological and pathopsychological features of cognitive and emotional disorders in patients with type II diabetes mellitus.

**Objectives:**

In the course of the study, a comprehensive examination of 109 patients with type II diabetes mellitus of moderate to severe forms of disease (52 females and 57 males), aged 35.9 ± 10.1 years was conducted.

**Methods:**

MMSE, Addenbrooke’s Cognitive Examination, Luria’s Memory Words Test-Revised, Symptom Check List-90-Revised, HAM-A, HAM-D, The State-Trait Anxiety Inventory Scale. It has been established that patients with diabetes mellitus have mild to moderate cognitive impairment, which is manifested by a reduction in verbal memory, a decrease in the speed of counting operations, minor difficulties in orientation and a slight drop in the perceptual and diagnostic sphere, a decrease in concentration and memorization of the information received, and a pronounced reaction of mental fatigue.

**Results:**

Presence of mild (51.2% of men and 49.8% of women) or moderate (49.8% and 50.3%, respectively) cognitive impairment according to MMSE and Addenbrooke’s Scale; decreased ability to concentrate and impaired working memory in patients with type II diabetes mellitus was noted.

Emotional disorders in patients with diabetes mellitus are represented by anxiety (33.4% of men and 35.2% of women), depression (26.6% and 33.1% of patients, respectively), astheno-hypochondriacal (27.3% of men and 19.1% of women), and hysteroform (12.7 and 12.6%, respectively) variants of psychopathological syndromes.

**Conclusions:**

A comprehensive, personalized system for correction of cognitive and emotional disorders associated with diabetes mellitus has been developed and tested. System comprises four stages: diagnostic, therapeutic, rehabilitation and dynamic monitoring. It employs a range of therapeutic modalities, including pharmacotherapy, psychotherapy and psychosocial programmes.

The implementation of the developed system for correction and prevention of emotional and cognitive disorders in patients with type II diabetes mellitus contributes to an improvement in the quality of medical care for patients with type II diabetes mellitus, as well as an improvement in their quality of life and psychosocial functioning.

**Disclosure of Interest:**

None Declared

